# High-Throughput GPCRome Screen of Pollutants Reveals the Activity of Polychlorinated Biphenyls at Melatonin and Sphingosine-1-phosphate Receptors

**DOI:** 10.1021/acs.chemrestox.3c00388

**Published:** 2024-01-31

**Authors:** Joshua C. Wilkinson, Hans-Joachim Lehmler, David L. Roman

**Affiliations:** † Department of Pharmaceutical Sciences and Experimental Therapeutics, College of Pharmacy, University of Iowa, Iowa City, Iowa 52242, United States; ‡ Department of Occupational and Environmental Health, College of Public Health, University of Iowa, Iowa City, Iowa 52242, United States; § Interdisciplinary Graduate Program in Neuroscience, University of Iowa, Iowa City, Iowa 52242, United States; ∥ Interdisciplinary Graduate Program in Human Toxicology, University of Iowa, Iowa City, Iowa 52242, United States; ⊥ Iowa Neuroscience Institute, Roy J. and Lucille A. Carver College of Medicine, University of Iowa, Iowa City, Iowa 52242, United States

## Abstract

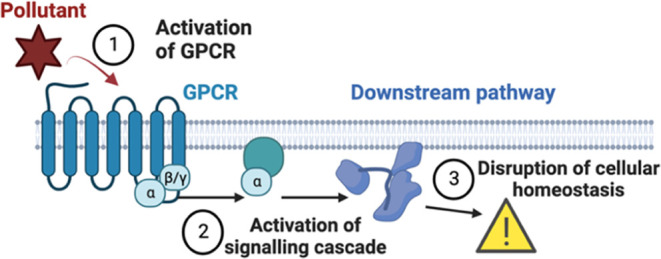

Exposure to environmental pollutants is linked to numerous toxic outcomes, warranting concern about the effect of pollutants on human health. To assess the threat of pollutant exposure, it is essential to understand their biological activity. Unfortunately, gaps remain for many pollutants’ specific biological activity and molecular targets. A superfamily of signaling proteins, G-protein-coupled receptors (GPCRs), has been shown as potential targets for pollutant activity. However, research investigating the pollutant activity at the GPCRome is scarce. This work explores pollutant activity across a library of human GPCRs by leveraging modern high-throughput screening techniques devised for drug discovery and pharmacology. We designed and implemented a pilot screen of eight pollutants at 314 human GPCRs and discovered specific polychlorinated biphenyl (PCB) activity at sphingosine-1-phosphate and melatonin receptors. The method utilizes open-source resources available to academic and governmental institutions to enable future campaigns that screen large numbers of pollutants. Thus, we present a novel high-throughput approach to assess the biological activity and specific targets of pollutants.

## 1 Introduction

Many chemicals for industrial and consumer applications inevitably pollute our food, water, and air.^1^ Understanding the biological impact of human exposure to pollutants is necessary to gauge their threat to our health and inform regulatory decisions. A lack of proactive measures taken to identify and assess the toxicity of pollutants can result in considerable public health concerns—exemplified by historic pollutants such as polychlorinated biphenyls (PCBs).^2^

While adverse health outcomes have been established for many pollutants, determining their specific targets and mechanisms remains challenging. For example, the family of PCBs, legacy pollutants marked as hazardous and banned decades ago, is still subject to much research and new findings.^3−8^ Moreover, the flow of new pollutants is relentless, leaving toxicologists inundated with compounds to investigate. Therefore, the ability to simultaneously evaluate large numbers of pollutants for their potential impact on biological systems is essential. Consequently, high-throughput screening (HTS) techniques enabling rapid and broad assessment of pollutant activity have become of great interest to toxicologists, as seen with the “Toxicology in the 21st Century” (Tox21) research consortium.^9,10^ In this work, we leverage G-protein-coupled receptors (GPCRs) and contemporary HTS resources in a novel approach to assess pollutant activity.

A superfamily containing hundreds of unique membrane receptors, GPCRs initiate signaling cascades for many physiological processes. Mechanisms of GPCR activation by ligands and the subsequent activation of distinct signaling pathways are reviewed in detail elsewhere.^11,12^ Briefly, GPCRs respond to changes in the extracellular environment by recognizing myriad ligands from macromolecules to ions,^13^ initiating cellular responses, and ultimately impacting systems from the cellular to the organismal level. The cellular responses initiated by ligand–GPCR binding are predominantly transduced via the activation of distinct G-protein members. Once active, G-proteins interact with various downstream effector proteins that modulate signaling molecules (e.g., second messengers such as cAMP and Ca^2+^) and, ultimately, the cellular response.^11^ Canonically, to terminate receptor signaling, activated GPCRs are phosphorylated by GPCR kinases (GRKs), followed by the recruitment of β-arrestin proteins. Generally, the recruitment of β-arrestin to an activated GPCR is considered an off-switch. However, mechanisms exist where β-arrestin functions in nontraditional signaling pathways.^14−16^

Given the manifold role GPCRs play in maintaining physiological equilibria and the broad disruption of these equilibria by pollutants, it is reasonable to predict that pollutant–GPCR interactions exist and contribute to the adverse outcomes associated with pollutant exposure. Intuitively, reports in the literature have implicated GPCRs and GPCR signaling events as targets of pollutant toxicity.^17−19^ However, few studies exist that directly examine pollutant–GPCR interactions. These studies are of limited scope, investigating select pollutants and receptors in detail, which leaves a vast majority of the GPCR landscape unexplored. We hypothesize that significant relationships between established pollutants and GPCRs remain to be discovered and that a broad-ranging approach will show novel pollutant–GPCR activity.

The paucity of research focused on the activity of pollutants at GPCRs (collectively, the GPCRome) is unsurprising due to a lack of resources and tools, which makes holistic studies difficult, time-consuming, and costly. Two modern techniques and tools that lower this barrier are the resources PRESTO-Tango^20^ and TRUPATH.^21^ An illustration and detailed description of the resources are provided in the Supporting Information (Figure S1). Briefly, PRESTO-Tango utilizes the TANGO (transcriptional output following arrestin translocation) system, where the recruitment of β-arrestin to the GPCR of interest causes the expression of luciferase, a light-producing enzyme.^22^ TRUPATH utilizes G-protein subunits labeled for bioluminescence resonance energy transfer (BRET) experiments to examine the activation of specific G-proteins. In combination, these resources enable the rapid and efficient screening of a GPCR library containing 314 unique receptor members and the subsequent investigation into the downstream signaling events of individual receptors. Further adding to their utility, both resources are open source (available to academic and nonprofit institutions), modular in design, and amenable to HTS. While PRESTO-Tango and TRUPATH are intended for drug discovery and detailed pharmacology, in this work, we show for the first time that they also have potential as tools for high-throughput toxicological investigations.

## 2 Materials and Methods

### 2.1 Preparation and Handling of DNA Plasmids

GPCR-tango and Gα,β and γ plasmids were purchased as bacterial stocks from Addgene (PRESTO-Tango #1000000068, and TRUPATH #1000000163); MTNR1B and S1PR4 cDNA receptor plasmids used for the Gαβγ dissociation assays were obtained from the cDNA Resource Center (www.cdna.org). For TANGO plasmids used in the primary screen, bacterial stabs were directly transferred to Luria Broth supplemented with 50 μg/mL of ampicillin and prepared via QIAprep Spin Miniprep Kit (QIAGEN, #27104) before evaluating the concentration and purity by measuring OD_260/280_ (Synergy2, BioTek.). To prepare library plates for the primary screen, plasmids were diluted to 30 ng/μL in 10 mM tris-HCl, pH 8.5, and aliquoted to their respective wells in 384-well polypropylene plates (ThermoFisher, #264573). All plasmids used in the subsequent dose–response and validation assays were prepared similarly with Wizard Plus Midiprep (Promega, Madison, WI). The identity of the receptor plasmids considered hits was confirmed via Sanger sequencing (University of Iowa, Genomics Core).

### 2.2 Cell Culture and Maintenance

HEK293T cells (ATCC, CRL-3216) were maintained in complete media comprised of Dulbecco’s modified Eagle’s media (DMEM) supplemented with 10% heat-inactivated fetal bovine serum and +1% penicillin–streptomycin. HTLA cells, a gift from the Barnea laboratory, were maintained similarly but with the addition of 100 μg/mL of hygromycin B and 10 μg/mL of puromycin. Several batches of frozen cells were used throughout experiments, with no more than a 10-passage difference in their starting number. Once thawed, cells were used within 3–20 passages.

### 2.3 Primary Screen and TANGO Assay

In the primary screen, TANGO assays were conducted using a “batch transfection” to facilitate high throughput. On day 1, cells were detached via trypsin–EDTA solution, resuspended in complete growth media, and seeded at 10,000 cells/well in poly-d-lysine-coated 384-well cell culture plates (Millipore Sigma-Aldrich, #Z759279) at a final volume of 35 uL. On day 2, GPCR-Tango DNA library plates and Transit-2020 (Mirus Bio) were used to form a batch transfection plate. Transfection complexes were prepared in a 384-well polypropylene plate (Thermo Scientific, #264573) at a final concentration of 10 ng/μL of DNA, with a ratio of 1 μg of DNA to 3 μL of transfection reagent. Transfection complexes were incubated for 20 min before transferring 3 μL to cell plates using the liquid handling station (Microlab Star, Hamilton), resulting in test plates transfected with 96 receptors in quadruplicate. On day 3, to reduce the potential impact of serum components on receptor activity, media was removed and replaced with 30 μL of complete growth media diluted 1:10 in Opti-MEM (Thermo Scientific #31985070). On day 4, 10 μL of 20 μM test compound (5 μM final) and vehicle control were added to test plates, both in duplicate for each receptor. On day 5, 30 μL of cell supernatant was carefully removed before the addition of 30 μL of Steady-Glo luciferase reagent (Promega) diluted 1:10 in cell culture media. After 30 min of incubation with Steady-Glo, relative light units (RLUs) were measured (Synergy2, Biotek). Pollutant–receptor pair follow-up experiments for the pollutant–receptor hit confirmation were conducted identically to the primary screen. The method for determining and validating pollutant–GPCR pairs as hits is described in the Supporting Information.

All subsequent TANGO assays were conducted similarly except transfection complexes were added to wells using the Mantis liquid handling platform (Formulatrix). In experiments that include antagonists, media was supplemented with the indicated antagonist or control for ∼60 min before compound addition. RLU values are shown as % change relative to the vehicle controls (i.e., basal activity) of individual experiments to correct for variability between experiments.

### 2.4 G-Protein Dissociation Assays

Gαβγ dissociation assays were performed as described by the original authors with minimal modifications.^23^ Briefly, HEK293T were seeded in 12-well plates and transfected 4–8 h after reaching ∼80% confluence with plasmid constituents (i.e., *GPCR*X, Gα_X_-RLuc8, GFP2-GγX, and GβX) at a total amount of 250 ng of DNA. All plasmids were kept at an equal 1:1:1:1 ratio, and transfection complexes were formed identically to the primary screen. Twenty-four h post transfection, cells were washed, detached using a Versene–EDTA solution (Gibco, Cat no. 15040066), spun down (450*g* × 2 min), resuspended in complete media diluted 1:10 in Opti-MEM, and 35 μL of 650 cells/μL (∼23,000 cells) were plated in poly-d-lysine-coated 384-well plates. The following day, using the Microlab Star (Hamilton), media was aspirated from wells and replaced with 15 μL of the test compound in drug buffer (20 mM HEPES, 0.5 mg/mL BSA, PH 7.5) or no compound control. Vehicle and reference ligand controls were distributed across rows to account for any possible drift or edge effects. Following the addition of compound or vehicle, 5 μL of 30 μM Prolume Purple (NanoLight Technologies Cat no. 369) dissolved in drug buffer was added to cells (7.5 μM final). Plates were equilibrated in the plate reader (Biotek, synergy2) for ∼30 min before measuring luminescence with the emission filters 440± 30 and 516 ± 20 nm for Rluc8 and GFP signal, respectively. BRET ratios were determined by taking the ratio of RLUs (relative light units) from GFP2 to RLUs from the Rluc8. BRET ratios of vehicle controls are subtracted from the tested conditions to determine the net change in the ratios (ΔBRET). To represent activation as an increase in the BRET signal relative to the vehicle, all ΔBRET values are represented as their negative value. In experiments where data are normalized as % response, ΔBRET values of the vehicle controls are set to 0%, and ΔBRET values of the reference ligand are set to 100%. All ΔBRET and % response values were determined from individual experiments using their respective control groups.

### 2.5 Data Analysis

All calculations of statistical significance were accomplished using GraphPad Prism 10. *P* values for the primary screen and the repeated hit confirmation experiments were determined via multiple unpaired Student’s *t* tests, with no corrections for comparisons. For comparisons of PCB congener activity at sphingosine and melatonin receptors, data were compared to vehicle control using one-way ANOVA with Dunnett’s post-test for multiple comparisons. For experiments of PCB congeners with the panel of Gα proteins, compound groups were compared to Gα proteins via unpaired *t* test Holm–Sidak post-test for multiple comparisons. Curves for concentration–response experiments were fit using a 4-parameter logistic regression. In all experiments, comparisons with *P* < 0.001 compared to vehicle control are marked with an asterisk.

### 2.6 Chemicals

The Iowa Superfund Research Program (ISRP) supplied the pollutants used in the pilot study. PCBs and metabolites were synthesized and authenticated by the ISRP Synthesis Core as previously described.^24−26^ A description of pharmacological agents and pollutants can be found in the Supporting Information. Chemical identifiers of pollutants are shown in the Supporting Information (Table S1).

## 3 Results and Discussion

### 3.1 Validation of Primary Screen

The PRESTO-Tango platform consists of 314 nonolfactory *GPCRX*-TANGO plasmids (i.e., the druggable-GPCRome), including well-characterized receptors and poorly understood orphan receptors.^20^ Within the library, the activity of individual receptors is diverse and contextually dependent. Basal activity and the magnitude of activation of GPCRs contained in the library can span orders of magnitude depending on the receptor(s) in question—a fundamental characteristic of GPCR biology.^20,27^ Even for the same receptor, activation levels and signal windows exhibited can vary depending on the ligand. In short, each GPCR must be considered its own system, and comparing the magnitudes of activation by a ligand across the entire GPCRome library is inappropriate.

**Figure 1:**
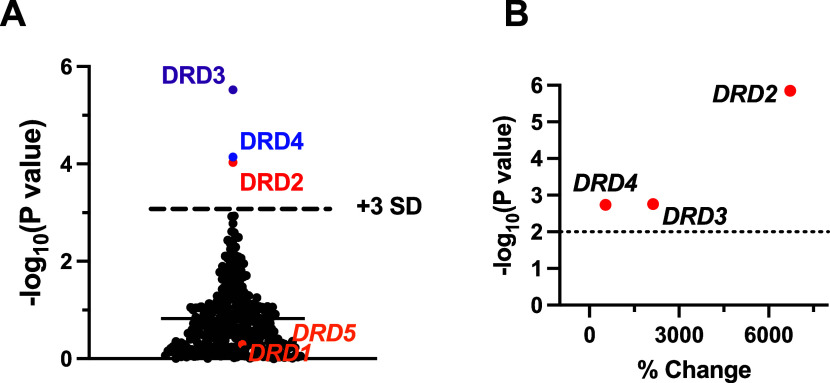
Validation of screening design using (−)-quinpirole. (A) Results of GPCR library screen from treatment with 100 nM (−)-quinpirole. The three D2-like dopamine receptors (DRD2, DRD3, and DRD4) were marked above the set threshold (mean +3 SD). (B) Volcano plot of repeated hit confirmation experiments for marked receptors (*n* = 3, triplicate). Data are shown as difference from vehicle (% change) on the *X*-axis and statistical significance on the *Y*-axis (−log (*P* value)). In the repeated confirmation experiments, ligand–receptor pairs with *P* <. 01 (i.e., −log(*P* value) > 2) are considered hits.

In our application, we account for the inherent variability by discerning pollutant–receptor pairs based on their statistical significance (i.e., *P* value) relative to the control population rather than their difference in magnitude. This approach values individual ligand–receptor pairs across the entire library with respect to only the receptor in question and has little bias to the magnitude of change. Additionally, when ranking ligand–receptor pairs, *P* values are compared only with those of the same test compound, which would help to control for any nonspecific impact (e.g., vehicle differences, compound cytotoxicity, etc.). Ultimately, we chose this analysis method to (1) help correct inter- and intrareceptor variabilities and (2) enable the detection of ligand–receptor interactions, which may only show a small but consistent response in activity. To validate our method, the selective D2-like dopamine receptor agonist (−)-quinpirole^28^ was screened at the GPCRome library as a control compound. Treatment with 100 nM (−)-quinpirole identified the family of D2-like dopamine receptors (*DRD2, DRD3, and DRD4*) as ligand–receptor pairs for hit confirmation, while the D1-like dopamine receptors (*DRD1, DRD5*) were unaffected by the ligand (Figure 1A). The D2-like receptors were confirmed as hits in follow-up validation experiments (Figure 1B). The results from this control experiment validate our approach and statistical method for identifying specific ligand–receptor interactions among the 314 unique GPCRs.

### 3.2 Identification of PCB Congeners as Ligands for Melatonin and Sphingosine Receptors

**Figure 2:**
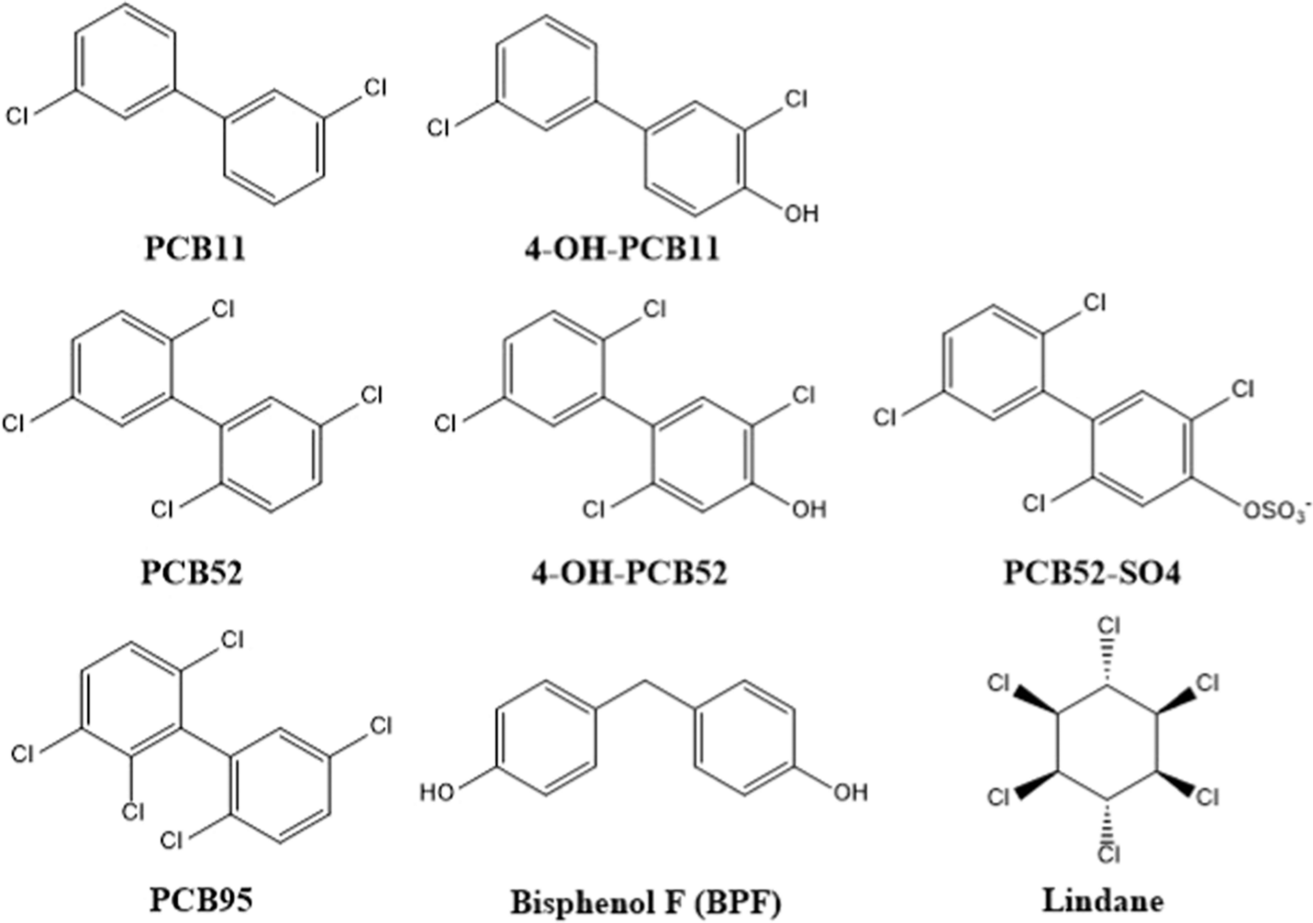
Structures and names of environmental pollutants screened in the GPCR library.

To test the ability of the GPCRome screen to discover pollutant–receptor pairs, eight environmental pollutants were interrogated in the GPCR library. The pollutants evaluated were three polychlorinated biphenyl (PCB) congeners (PCB11, PCB95, and PCB52), three PCB metabolites (4-OH-PCB11, 4-OH-PCB52, and PCB52-SO4), bisphenol F (BPF), and lindane. Pollutants were chosen based on established interest in their mechanisms of toxicity and their structural similarities (Figure 2)

Briefly, PCBs are recognized to cause diverse health outcomes, mainly including neurotoxicity and neurodevelopment disorders,^29^ immune dysfunction,^30^ endocrine disruption,^31^ and reproductive toxicity.^32^ The PCBs chosen in this study include prominent legacy PCBs (PCB52 and PCB95), nonlegacy PCBs of emerging concern (PCB11), and relevant metabolites, all with particular interest for their implications in neurotoxicity and neurodevelopment disorders.^8,29,33^ Bisphenols are a class of pollutants characterized by their endocrine-disrupting function, best illustrated by the notorious member, bisphenol A (BPA).^34^ BPF, a structural analog of BPA, is an emerging bisphenol pollutant with increasing evidence for broad toxicity ranging from metabolic^35^ to reproductive functions.^36^ Lindane is a pesticide with restricted or banned use in most nations due to its established harm that ranges from neurotoxicity to cardiac arrhythmias.^37^ Collectively, these pollutants evaluate the capability of our screening procedure to determine specific activity among structurally related pollutants (Figure 2) with meaningful implications.

**Figure 3:**
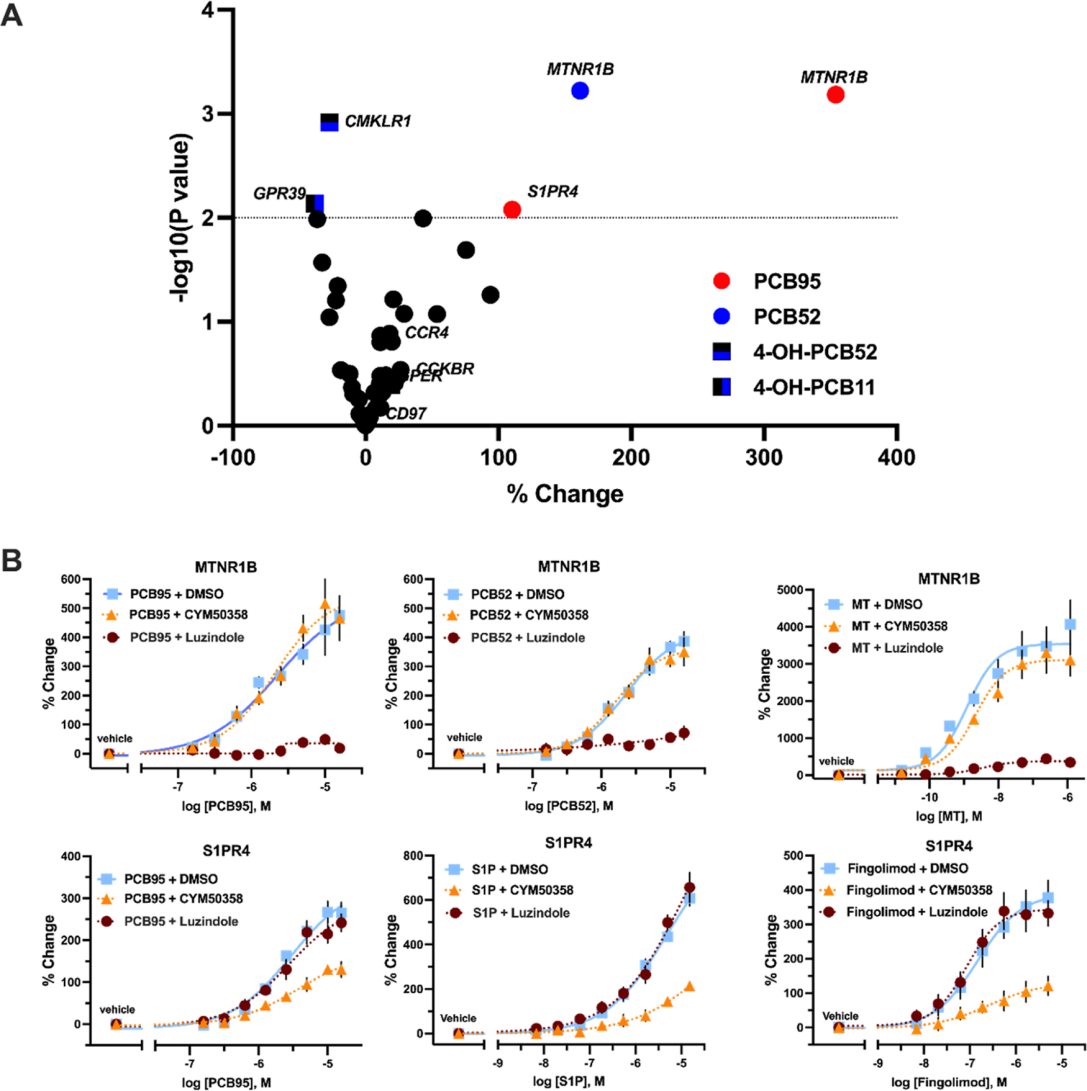
Hits identified from pollutant pilot screen (*n* = 3). (A) Volcano plot of pollutant–GPCR pairs investigated (5 μM) for hit confirmation. Pollutant–GPCR pairs with a −log (*P* value) >2 (dotted line) are considered hits. (B) Concentration–response experiments of identified contaminant–receptor pairs, dosed at receptors preincubated with S1PR4 antagonist (2 μM, CYM50358), MTNR1B antagonist (500 nM luzindole), or DMSO control.

Overnight treatment with 5 μM pollutants resulted in 49 ligand–receptor pairs marked above the threshold for hit confirmation. The distribution of pollutant–receptor pairs for each compound and the list of pairs marked for hit confirmation can be found in the Supporting Information (Figure S2 and Table S1). Follow-up confirmation experiments identified five pollutant–receptor pairs as hits (Figure 3A). Of these five ligand–receptor pairs marked as hits, three showed appreciable concentration–response curves: PCB95 at sphingosine-1-phosphate receptor 4 (*S1PR4*) and both PCB95 and PCB52 at melatonin receptor 1B (*MTNR1B*). Concentration–response experiments of the PCB congeners at S1PR4 and MTNR1B receptors showed a concentration-dependent increase in β-arrestin recruitment relative to the no compound control, with activity in the low-micromolar range (Figure 3B). Furthermore, preincubation with S1PR4 antagonist, CYM50358,^38^ and melatonin receptor antagonist, luzindole,^39^ blocked activity from PCBs specific to the receptor. Known ligands, melatonin (MT), sphingosine-1-phosphate (S1P), and fingolimod,^40^ were used parallel as positive controls to validate receptor pharmacology. The remaining two pollutant–receptor pairs were considered false positives, as no evident activation was observed in concentration–response experiments (Figure S3), and their activity in the confirmation experiments was incongruent with their activity from the primary screen.

The number of validated hits from ligand–receptor pairs marked for confirmation (3 of 49) illustrates the nature of high-throughput methodologies that often result in small proportions of initial hits being validated in subsequent experiments. In our iteration of the screen, the threshold for hit confirmation was set as three standard deviations above the mean log *P* value. While a more stringent cutoff, for example, log *P* > 2.5, could result in fewer false positives, this would have also eliminated PCB52-*MTNR1B*, which was later validated as a hit. Furthermore, it was observed that several serotonin (5-hydroxytryptamine; 5-HT) receptors were clustered around the threshold for bisphenol F (BPF) but missed the cutoff by narrow margins (Figure S4). Briefly, BPF and structurally related bisphenol compounds are implicated in various aspects of serotonin signaling.^41,42^ Consequently, the pollutant–receptor pairs of BPF and serotonin receptors could represent false negatives. Thus, a balance must be struck between the possibility of a greater number of false positives vs too high stringency, which could result in false negatives.

### 3.3 PCB Activity at Melatonin and Sphingosine Receptors Is Specific to Congener and Receptor

**Figure 4:**
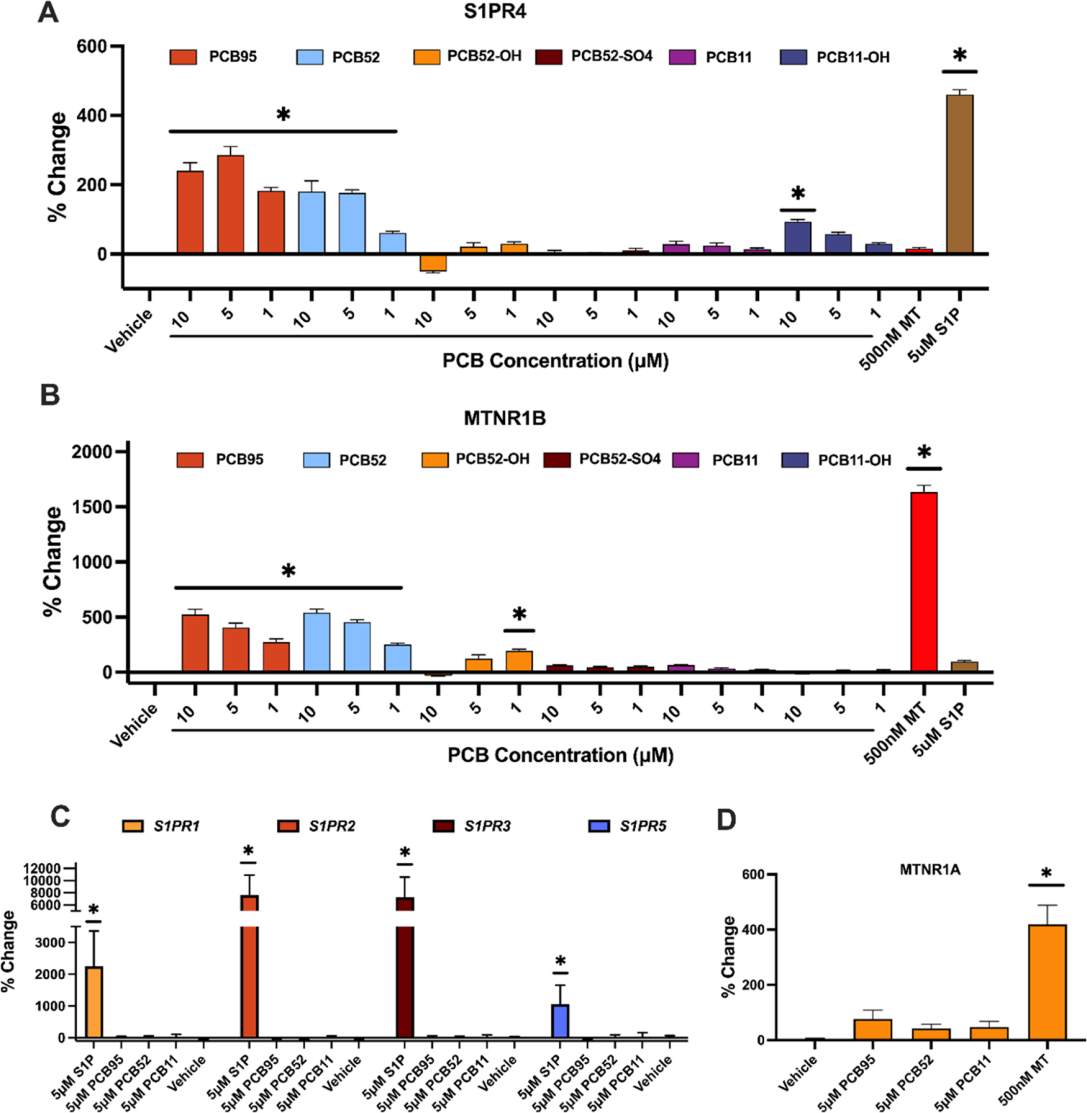
Activity of PCBs at sphingosine-1-phosphate and melatonin receptors is selective for PCB congeners and receptor family members. Treatment with three concentrations of PCB congeners and metabolites at (A) sphingosine-1-phosphate receptor 4 (S1PR4) and (B) melatonin receptor 1b (MTNR1B). (C) 5 μm PCB parent congeners at sphingosine-1-phosphate receptor isoforms 1,2,3, and 5 (S1PR1–5), and (D) melatonin receptor 1a (MTNR1A). Values are shown as % change relative to vehicle control, data presented as mean ± SEM (*n* = 3, quadruplicate). (*) Significantly different from vehicle control at *p* < 0.001; one-way ANOVA with Dunnett’s post-test for multiple comparisons. (MT = melatonin; S1*P* = sphingosine-1-phosphate).

Humans express multiple receptors, recognizing melatonin and sphingosine-1-phosphate as their primary endogenous ligands. Melatonin has two receptor subtypes,^43^*MTNR1B* and the melatonin receptor 1A (*MTNR1A*), while sphingosine-1-phosphate has five receptor isoforms^44^ (*S1PR1–5*). All members of the receptor families were included in the receptors screened. However, only the receptor members S1PR4 and MTNR1B were identified as targets for distinct PCBs demonstrating specific activity for PCB congeners and receptor subtypes/isoforms. To affirm these findings, PCB congeners were tested at S1PR4 and MTNR1B at three concentrations: 10, 5, and 1 μM (Figure 4A,B). Concentration-dependent activity was observed for PCB95 and PCB52 at both receptors. Additionally, the 4-OH-PCB52 metabolite had significant activity at *MTNR1B*, with a similar magnitude to that of the parent congener at 1 μM, and the 4-OH-PCB11 metabolite had a concentration-dependent trend in activation at *S1PR4*. No significant activity was detected for PCB52-SO4 or PCB11 at either receptor. Next, we evaluated if PCB parent congeners showed activity at the other melatonin and sphingosine receptor family members (Figure 4C,D). Interestingly, none of the PCB parent congeners tested (5 μM) at S1PR1,2,3,5 and MTNR1A had significant activation of the receptors.

These findings indicate that the activity of PCB congeners on sphingosine-1-phosphate and melatonin receptor families is specific to both the congener and the receptor family members. Results show that activity is limited to the S1PR4 and MTNR1B receptors, with activity predominately attributed to PCB95 and PCB52 and, to a lesser extent, to the hydroxylated metabolites. These results demonstrate the capability of our screening approach to discern specific pollutant–receptor pairs from compounds of related structure and biological targets of conserved function.

Identifying activity from PCB95 and PCB52, at S1PR4 and MTNR1B, but not PCB11, suggests that structural differences contribute to the specific activity of PCB congeners. While similar in chemical composition, PCB congeners can be classified by the orientation of their phenyl rings. PCBs can also be described as dioxin-like (DL) or nondioxin-like (NDL). The structural differences between DL and NDL-PCBs are known to contribute to divergent biological activity and toxicity.^45^ DL-PCBs are well-known to activate the aryl hydrocarbon receptor (AhR), and this action is considered a primary mechanism for their adverse outcomes. The biological activity of NDL-PCBs is less characterized, having more diverse putative targets, such as the constitutive androstane receptor (CAR) and the pregnane-xenobiotic receptor (PXR).^46^ PCB95 and PCB52 have chlorines at the ortho positions of the ring junctions that constrain the biphenyl rings in a more rigid, noncoplanar orientation,^47^ while PCB11 lacks ortho-substituted chlorines. We postulate that the more prominent activity of PCB95 and PCB52 could be due to these ortho-substituted chlorines and their NDL structure. Future investigations that examine differently substituted PCB congeners at S1PR4 and MTNR1B could provide valuable insight into the structural activity relationships.

### 3.4 Orthogonal Validation of PCB Activity at MTNR1B and S1PR4 with G-Protein Dissociation Assays

Canonically, activated GPCRs transduce their signal via the dissociation of Gαβγ heterotrimer complexes, where the dissociated (i.e., active) Gα and Gβγ subunits act on downstream effector proteins to produce a cellular response.^11^ Several families of Gα subunits contain over a dozen distinct members that GPCRs can activate, dictated by the receptor’s structure and the conformational change specific to ligand binding.^12^ The TANGO assay measures the end point at the receptor level via β-arrestin recruitment, which can be independent of G-protein activation. While measuring activity at the receptor level enables a homogeneous output for receptors coupled to different cellular pathways, it cannot describe what downstream signaling pathways are activated. Thus, additional systems are needed to confirm pollutant activity at receptors beyond β-arrestin recruitment.

**Figure 5:**
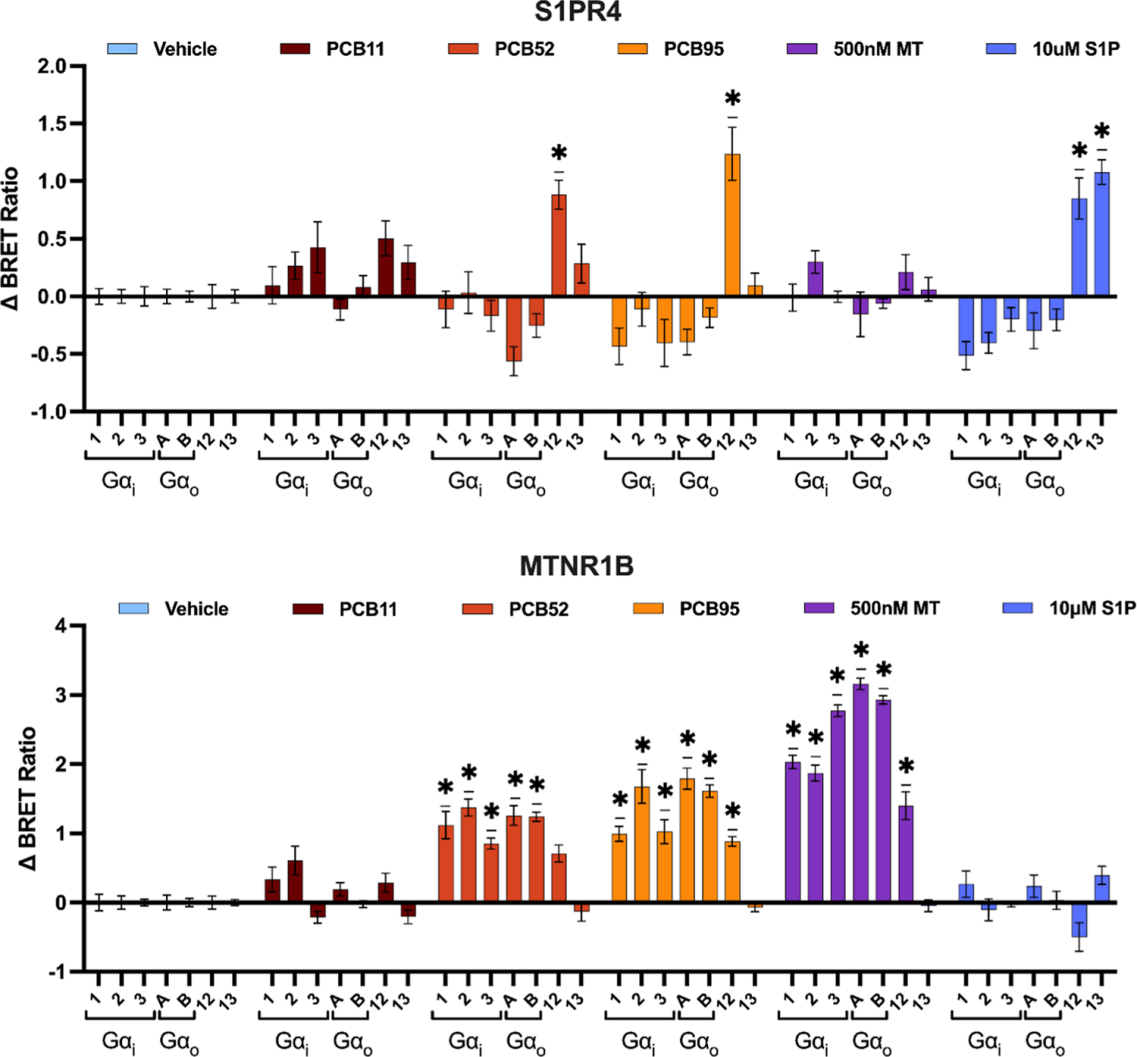
PCB activity (10 μM) on the dissociation of Gα_i/o_ and Gα_12/13_ protein subunits at S1PR4 and MTNR1B. Values are shown as the net change in the BRET ratio from the vehicle control (ΔBRET ratio). Data represent mean ± SEM (*n* = 4, duplicate). (*) Significantly different from vehicle control at *p* < 0.001; unpaired *t* test Holm–Sidak post-test for multiple comparisons (MT = melatonin; S1*P* = sphingosine-1-phosphate).

To validate the interactions identified from the primary screen in an orthogonal assay and gain insight into potential activation mechanisms, we examined parent PCB congeners at S1PR4 and MTNR1B to trigger specific G-protein α (Gα) subunit dissociation. Using the BRET assays (i.e., TRUPATH), PCB activity at S1PR4 and MTNR1B was tested with the Gα_i/o_ and Gα_12/13_ families, as these are the primary reported signal transducers for melatonin^48^ and S1P^49^ receptors (Figure 5).

Congruent with the prior experiments, PCB95 and PCB52 (10 μM) had significant activity for G-protein subunit activation at both receptors, while PCB11 had no significant activity at either receptor. Control experiments using the dopamine D2 receptor (Figure S5) show that the change is not a product of nonspecific events (e.g., compound interference with the BRET signal). These results orthogonally validate the findings from the β-arrestin experiments and further establish PCB activity at S1PR4 and MTNR1B.

Interestingly, selective trends in the activation of Gα proteins by PCBs were detected from the BRET experiments. Notably, at S1PR4, PCB52 and PCB95 show only activation of Gα_12_, while the native ligand S1P activates both Gα_12_ and Gα_13_ subunits. Furthermore, when melatonin was used as a reference for maximum activation, the relative activation from PCB95 and PCB52 was higher at MTNR1B-Gα_i2_ compared to that at MTNR1B-Gα_i1/3_. These results suggest that PCB congeners contain biased activity at the receptors and signal through distinct pathways. Concentration–response experiments were conducted for PCB95 and PCB52 at S1PR4-Gα_12/13_ and MTNR1B-Gα_i2/3_ to examine these relationships further.

**Figure 6:**
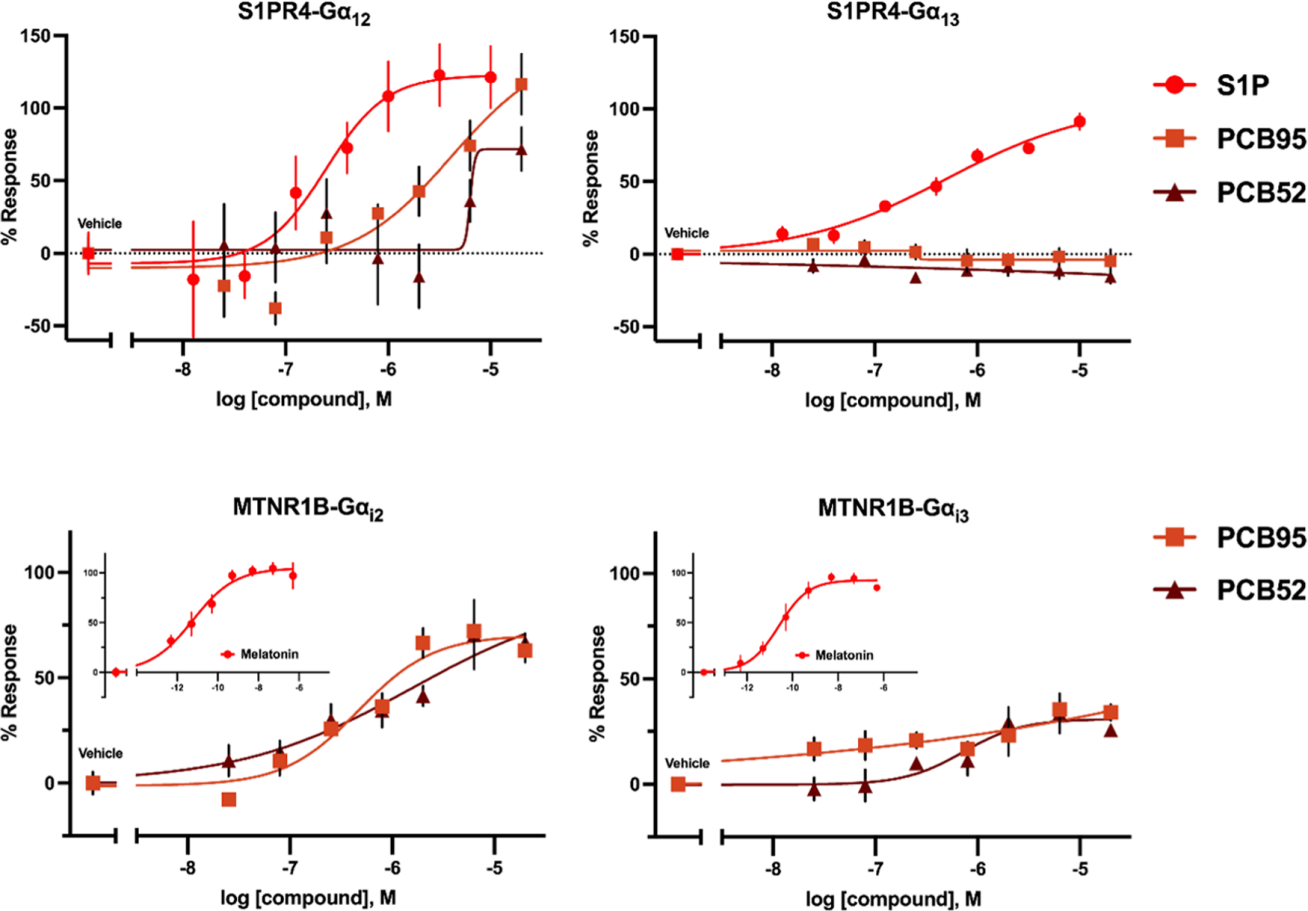
Concentration–response experiments of PCB congeners for G-protein dissociation with (top) S1PR4-Gα_12/13_ and (bottom) MTNR1B-Gα_i2/i3_ (*n* = 3, quadruplicate). Values are shown as % response, normalized to reference ligand (10 μM sphingosine-1-phosphate, S1P; or 500 nM melatonin, MT) and vehicle control. The concentration–response curve of the reference ligand for MTNR1B is imposed as a separate inlet on the graph. Data points represent the mean ± SEM.

**Figure 7:**
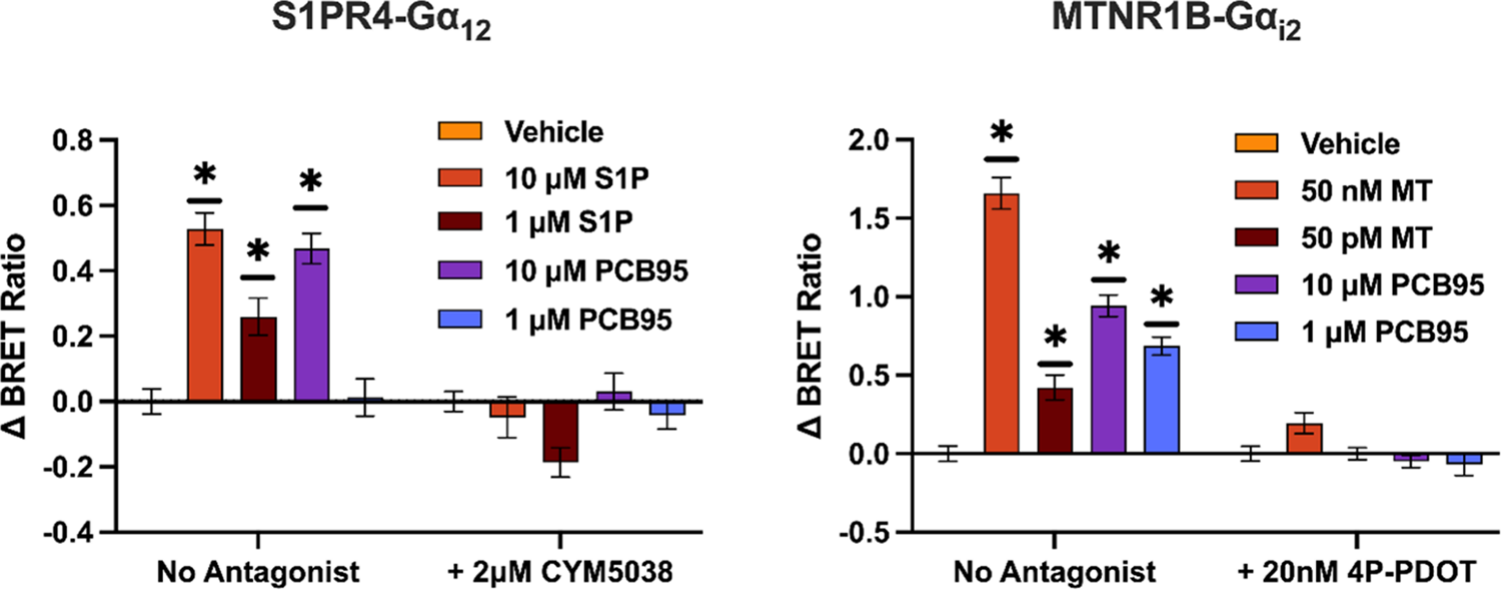
PCB congener activity on S1PR4-Gα_12_ and MTNR1B-Gα_i2_ in the presence or absence of receptor antagonist (*n* = 3, quadruplicate). Cells were treated with PCBs diluted in buffer containing S1PR4 antagonist (CYM5038), MTNR1B antagonist (20 nM 4P-PDOT), or no antagonist control. (*) Significantly different from vehicle control at *p* < 0.001; one-way ANOVA with Dunnett’s post-test for multiple comparisons (MT = melatonin; S1*P* = sphingosine-1-phosphate).

Affirming the initial observations, at S1PR4, PCB congeners exhibit activation solely for Gα_12_, while S1P activates both Gα_12_ and Gα_13_, and at MTNR1B, PCBs have a more pronounced response in activity for the Gα_i2_ subunit compared to that of Gα_i3_ (Figure 6). Additionally, these experiments show that PCB95 elicits a more robust response than PCB52, primarily at S1PR4. To confirm that the observed activity is receptor-mediated, we tested the ability of antagonists to block G-protein activation. Using PCB95 as a model system, cells were treated with PCB95 (10 and 1 μM) in the presence or absence of selective antagonists CYM50358 (2 μM) and 4P-PDOT^50^ (20 nM) at S1PR4-Gα_12_ and MTNR1B- Gα_ι2_, respectively. At each receptor, the antagonists significantly blocked the dissociation of Gα proteins from PCB exposure (Figure 7).

Together, results from the G-protein dissociation assays identify the PCBs as specific agonists for S1PR4 and MTNR1B. In this system, the activity of PCBs occurs after a brief exposure time (<30 min) and shows activity at low- and submicromolar concentrations. Additionally, results demonstrate that the activity is selective for PCB congeners and is biased toward specific signaling pathways. Future studies examining the relationships between PCBs and activated pathways in detail could provide new insight into contributing mechanisms and biological implications of PCB exposure. For example, altered g-protein signaling at MTNR1B has been correlated to metabolic diseases such as type II diabetes,^51^ and the cellular responses specific to Gα_12_ or Gα_13_ are vast.^52,53^

To the best of our knowledge, no studies have established PCB congeners as ligands for S1P or melatonin receptors, and we consider our findings novel. However, we believe that there is precedent literature that connects PCB activity to biological pathways proximal to the receptors and supports the significance of our findings. As a broad example, one set of studies shows that treatment with melatonin can protect against PCB-induced neurotoxicity.^54−59^ The protective role of melatonin in these studies is largely attributed to its antioxidant characteristics and its downstream action on gene expression.^60^ More specific examples are illustrated in select reports that establish PCB activity in cellular processes of circadian rhythm and sphingosine metabolism. These studies show that PCB exposure can alter essential genes regulating circadian rhythm^61,62^ and the metabolic pathway responsible for producing the S1P ligand.^63,64^ Interestingly, the studies link their findings to similar outcomes regarding PCB toxicity, notably the dysregulation of endothelial cell junctions from PCB exposure.^62,64^ The interrelationship of these activities is further supported by emerging reports that demonstrate crosstalk between melatonin and S1P signaling pathways, with implications at the receptor level.^65,66^ Collectively, these independent studies suggest that the activities from PCB95 and PCB52 at MTNR1B and S1PR4 reported here could be essential components in a broader mechanism underlying PCB toxicity. Future investigations into the biological activity of PCBs that consider the melatonin and sphingosine receptors could lead to more impactful discoveries.

Here, we demonstrate a novel HTS paradigm for assessing the activity of environmental pollutants. Advantageous for toxicological investigations, this approach can facilitate the interrogation of large numbers of pollutants at 314 diverse cellular targets. The value of this approach is highlighted in our pilot screen, where the previously unknown activity between specific PCB congeners and GPCRs was identified. Thus, this work provides fresh resources for examining the biological impacts of various environmental pollutants and compounds of concern.

## Supporting Information Available

Methods for determining pollutant–GPCR pairs, illustration and description of GPCR resources, distribution of pollutant–GPCR pairs in the primary screen, list of pollutant–GPCR pairs marked for hit confirmation, concentration–response curves of 4-OH-PCB52 and 4-OH-PCB11 false positives, BPF screening results with serotonin receptors highlighted, and test for nonspecific activity of PCBs on BRET experiments (PDF)
