# Supplementary material for: High-Throughput GPCRome Screen of Pollutants Reveals the Activity of Polychlorinated Biphenyls at Melatonin and Sphingosine-1-phosphate Receptors
Source: Chem Res Toxicol. 2024 Jan 31;37(2):439–49. doi: 10.1021/acs.chemrestox.3c00388 (PMC10880096; doi:10.1021/acs.chemrestox.3c00388)
Supplement: Supplementary file 1 — tx3c00388_si_001.pdf [file tx3c00388_si_001.pdf]

Supporting Information

High-Throughput GPCRome Screen of Pollutants Reveals the Activity of Polychlorinated Biphenyls at Melatonin and Sphingosine-1-Phosphate Receptors.

Joshua C. Wilkinson[1], Hans-Joachim Lehmler[2], David L. Roman[3*]

[1] Department of Pharmaceutical Sciences and Experimental Therapeutics, College of Pharmacy, University of Iowa, Iowa City, Iowa.
[2] Department of Occupational and Environmental Health, College of Public Health, University of Iowa, Iowa City, IA 52242, USA; Interdisciplinary Graduate Program in Neuroscience, University of Iowa, Iowa City, IA 52242, USA; Interdisciplinary Graduate Program in Human Toxicology, University of Iowa, Iowa City, IA 52242, USA
[3] Department of Pharmaceutical Sciences and Experimental Therapeutics, College of Pharmacy, University of Iowa, Iowa City, Iowa; Iowa Neuroscience Institute, Roy J. and Lucille A. Carver College of Medicine, University of Iowa, Iowa City, Iowa. Electronic address: david-roman@uiowa.edu.

Table of Contents:

Determining Pollutant-GPCR hits from the primary screen.	S2
Chemicals	S3
**Table S1** Chemical identifiers for pollutants	S4
**Figure S1** illustration of GPCR assays	S5
**Figure S2** Distribution of pollutant-GPCR pairs from primary screen	S6
**Table S2** list of pollutant-GPCR pairs marked for hit confirmation	S7-S8
**Figure S3** Concentration response curves of false positives	S9
**Figure S4** Screening data for BPF with serotonin receptors highlighted	S10
**Figure S5** Concentration response curves of PCBs at the dopamine receptor in BRET experiments	S11

Pollutant-receptor hit identification from the primary screen. To identify pollutant-receptor interactions in the primary screen, pollutant-receptor pairs were ranked by the statistical significance of the dosed (pollutant-containing) wells versus control wells. P values were calculated by comparing RLU values of the wells treated with compound (i.e., test population) against the RLU values of wells excluding the compound (i.e., control population) at the same receptor – resulting in sets of 314 P values for each compound. Next, P values were transformed to the negative log scale ($-1 * Log(P\ value)$), and the mean and standard deviation of the transformed P values were calculated for individual compound data sets. For each set of compound P values, a threshold was determined by taking three standard deviations above the mean, and any compound-receptor pair above this threshold is marked to be repeated in hit confirmation experiments. In the repeated confirmation experiments, compound-receptor pairs with a difference of $p < .01$ (i.e., $-\log_{10}(p\ value) > 2$) from vehicle control were considered hits and further validated in concentration-response experiments.

Chemicals and reagents

Powdered stocks of PCBs were resuspending in DMSO (Millipore Sigma), and BPF and Lindane in acetone (Millipore Sigma). Multiple aliquots of each pollutant were stored in sealed amber vials at -20 °C.

Pharmacological agents Sphingosine-1-phosphate d18:1 (S1P) (CAS 26993-30-6; Product no. 62570), fingolimod (CAS 162359-55-9; Product no. 11975), 4P-PDOT (CAS 134865-74-0; Product no. 17411), Luzindole (CAS 117946-91-5; Product no. 15998) were purchased from Cayman Chemical. CYM50358 (CAS 1314212-39-9; Product no. HY-136462) was purchased from MedChem Express. Melatonin (CAS 73-31-4; Product no. M5250) and (-)-quinpirole (CAS 85798-08-9; product no. Q102) were purchased from Millipore Sigma. Besides S1P, all stock solutions of pharmacological agents were dissolved in DMSO and stored as individual aliquots. Powdered S1P was reconstituted to a concentration of 1 mM in methanol supplemented with 0.1 M NaOH (Millipore Sigma), before storing as individual aliquots. Before use, methanol was evaporated under a stream of nitrogen, and S1P was reconstituted to 1 mM in buffered saline (DPBS) supplemented with 4 mg/mL BSA (Research product international, A30075)

Table S2 Chemical identifiers for pollutants

Abbreviation	IUPAC Name	InChIKey	CAS Number	DTXSID	Comptox Link
PCB95	2,2',3,5',6-pentachlorobiphenyl,	GXNNLIMMEXHBKV-UHFFFAOYSA-N	38379-99-6	DTXSID3038301	https://comptox.epa.gov/dashboard/chemical/details/DTXSID3038301
PCB52	2,2',5,5'-Tetrachlorobiphenyl	HCWZEPKLWVAEOV-UHFFFAOYSA-N	35693-99-3	DTXSID3038305	https://comptox.epa.gov/dashboard/chemical/details/DTXSID3038305
PCB11	3,3'-Dichloro-1,1'-biphenyl	KTXUOWUHFLBZPW-UHFFFAOYSA-N	2050-67-1	DTXSID70872817	https://comptox.epa.gov/dashboard/chemical/details/DTXSID70872817
4-OH-PCB52	2,5-dichloro-4-(2,5-dichlorophenyl)phenol	ZKDSNFDCQYBBIU-UHFFFAOYSA-N	51274-68-1	DTXSID10199272	https://comptox.epa.gov/dashboard/DTXSID10199272
PCB52-SO4	2,5-dichloro-4-(2,5-dichlorophenyl)phenylsulfate	N/A	N/A	N/A	N/A
4-OH-PCB11	2-chloro-4-(3-chlorophenyl)phenol	JOHAARQQFBMIOV-UHFFFAOYSA-N	53890-78-1	DTXSID10202159	https://comptox.epa.gov/dashboard/chemical/details/DTXSID10202159
BPF	4-[(4-hydroxyphenyl)methyl]phenol	PXKLMJQFEQBVLD-UHFFFAOYSA-N	620-92-8	DTXSID9022445	https://comptox.epa.gov/dashboard/chemical/details/DTXSID9022445
Lindane	(1R,2S,3r,4R,5S,6r)-1,2,3,4,5,6-Hexachlorocyclohexane	JLYXXMFPNIAWKQ-GNIYUCBRSA-N	58-89-9	DTXSID2020686	https://comptox.epa.gov/dashboard/chemical/details/DTXSID2020686

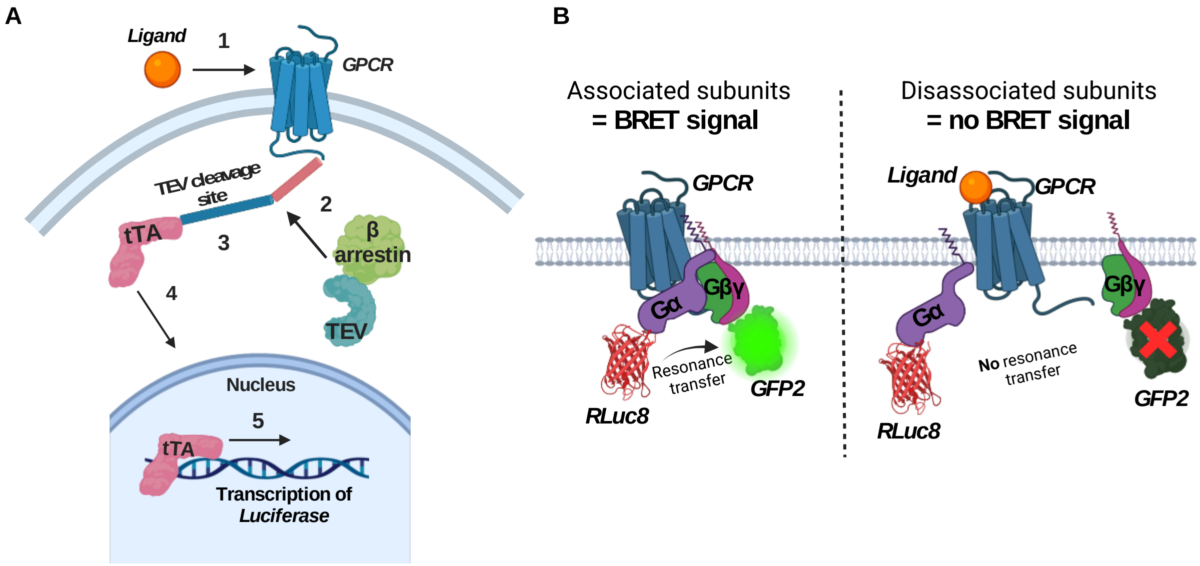

Figure S1. Schematics for GPCR activity assays. (A) TANGO assay, adapted from Kroeze et al. In the TANGO assay, the coding sequence for a GPCR of interest is expressed in HTLA cells via a "Tango-ized" plasmid (i.e., GPCR-TANGO). In this system, upon GPCR activation (1) a stably-expressed, engineered beta arrestin-TEV (Tobacco Etch Virus protease) fusion protein is recruited to the receptor (2), resulting in the cleavage at the TEV linkage site which has been engineered on the receptor, connecting a tTA transcription factor (3) this cleavage releases the tTA transcription factor, which then is translocated to the nucleus (4), where it initiates transcription of a luciferase gene (5). (B) In the TRUPATH assay, Gα-RLuc8 (Renilla Luciferase) and Gβ/Gγ-GFP2 (Green Fluorescent Protein) fusion proteins are used to measure the dissociation of specific Gα subunits. In the inactive state, the G-protein subunits form a heterotrimer, which brings the RLuc8 and GFP2 within proximity to facilitate BRET (Bioluminescent Resonance Energy Transfer) signal following luciferase (donor) activity that can excite the GFP acceptor (left panel). Upon activation, receptors influence the dissociation of Gα and Gβγ subunits, resulting in a decreased BRET signal due to the loss of proximity of the donor and acceptor pairs.

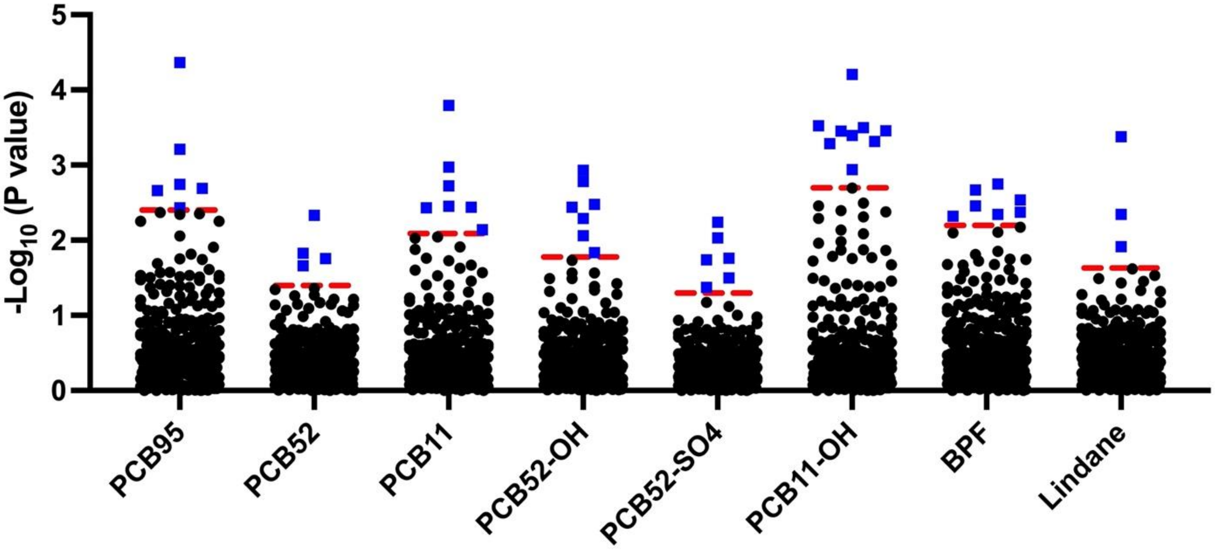

Figure S2 Distribution of ligand-receptor pairs marked for hit confirmation. Each data point represents a pollutant-receptor pair. Data points above the + 3 SD threshold (dashed line) are marked as blue squares.

Table 2. Ligand-receptor pairs above the threshold for hit confirmation from the primary screen.

**Compound** *(+3 SD Threshold)*	***Gene***	**Common Name**	**-Log (p value)**	**% Change**
**BPF** ***-Log P > 2.18***	*MRGPRD*	Beta-alanine receptor	2.7	61 ± 11
	CCR4	C-C motif chemokine receptor 4	2.7	46 ± 1
	CCKBR	cholecystokinin B receptor	2.5	87 ± 159
	GPER	G protein-coupled estrogen receptor 1	2.5	57 ± 30
	TACR1	tachykinin receptor 1	2.4	14 ± 19
	NTSR1	neurotensin receptor 1	2.3	43 ± 22
	CD97	adhesion G protein-coupled receptor E5	2.3	46 ± 95
**Lindane** ***-Log P > 1.64***	*GABBR1*	gamma-aminobutyric acid receptor 1	3.4	46 ± 124
	GPER	G protein-coupled estrogen receptor 1	2.3	-25 ± 13
	HCTR1	hypocretin receptor 1	1.9	-7 ± 39
**PCB95** ***-Log P >2.41***	*NPY4R*	neuropeptide Y receptor Y4	4.4	265 ± 124
	CCRL2	C-C motif chemokine receptor like 2	3.2	65 ± 254
	S1PR4	sphingosine-1-phosphate receptor 4	2.7	155 ± 47
	MTNR1B	melatonin receptor 1B	2.7	113 ± 131
	GPRC5D	N/A	2.7	53 ± 120
	HTR1B	5-hydroxytryptamine receptor 1B	2.4	69 ± 130
**4-OH-PCB11** ***-Log P >2.74***	*GPR174*	N/A	4.2	167 ± 35
	GPR142	N/A	3.5	139 ± 54
	GPR37	N/A	3.5	101 ± 53
	GPR65	N/A	3.5	112 ± 32
	GPR25	N/A	3.5	101 ± 23
	GPR132	N/A	3.4	58 ± 30
	GPR143	N/A	3.3	135 ± 114
	GPR182	N/A	3.3	56 ± 24
	GPR39	N/A	2.9	91 ± 50
**PCB11** ***-Log P >2.10***	*GPR18*	N/A	3.8	181 ± 58
	GRM4	glutamate metabotropic receptor 4	3.0	110 ± 16
	PRRP	Prolactin-releasing peptide receptor	2.7	104 ± 30
	OPRL1	opioid related nociception receptor 1	2.5	95 ± 16
	MRGPRF	MAS related GPR family member F	2.4	104 ± 10
	GPR75	N/A	2.4	79 ± 59
	GPR56	N/A	2.1	93 ± 10
**4-OH-PCB52** ***-Log P > 1.78***	*CMKLR1*	chemerin chemokine-like receptor 1	2.9	76 ± 97
	CCKAR	cholecystokinin A receptor	2.8	130 ± 103
	PTGFR	prostaglandin F receptor	2.5	167 ± 106

	GAL3	Galanin receptor 3	2.4	109 ± 62
	ADORA2A	adenosine A2a receptor	2.3	81 ± 55
	FFA1	Free Fatty acid receptor 1	2.1	92 ± 135
	GPR22	N/A	1.8	75 ± 8
**PCB52-SO4** *-Log P > 1.27*	*NPFF1*	Neuropeptide FF receptor 1	2.2	103 ± 1
	GRM2	glutamate metabotropic receptor 2	2.0	139 ± 191
	SUCNR1	Succinate receptor 1	1.8	-62 ± 2
	MCHR1	Melanin concentrating hormone 1	1.7	85 ± 1
	AVPR2	Arginine Vasopressin receptor 2	1.5	-34 ± 2
	FPR1	Formyl peptide receptor 1	1.4	75 ± 129
**PCB52** *-Log P > 1.39*	*MTNR1B*	melatonin receptor 1B	2.3	112 ± 16
	PTGIR	prostaglandin I2 receptor	1.8	86 ± 50
	ADRA1D	adrenoceptor alpha 1D	1.8	91 ± 26
	CALCRL	calcitonin receptor like receptor	1.7	74 ± 59

The -log (P value) value for hit confirmation (+3 SD threshold) is listed below each compound. The gene name and common name (if available) are shown for each receptor. For reference, the magnitude of activity is shown as the relative difference from the control (% change).

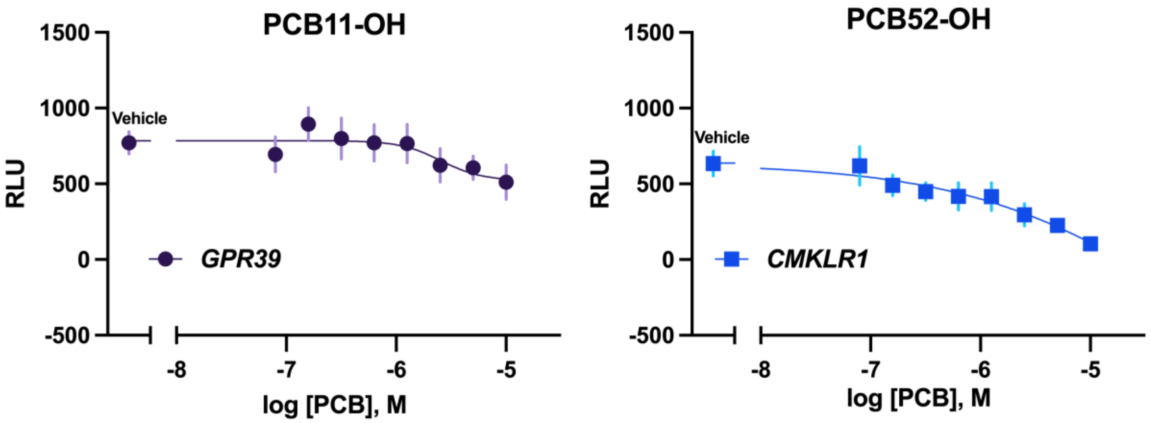

Figure S3 Concentration-response curves of pollutant-receptor pairs. Data points are shown as relative luminescence units (RLUs) mean +/- SEM, in duplicate (n = 3).

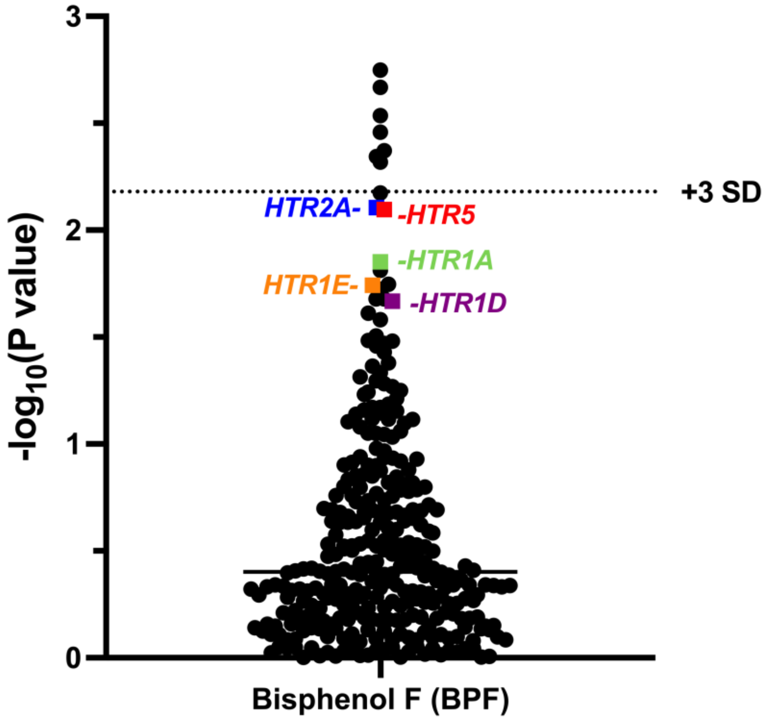

Figure S4 Initial screening data of BPF (5 μM), with serotonin receptors highlighted.

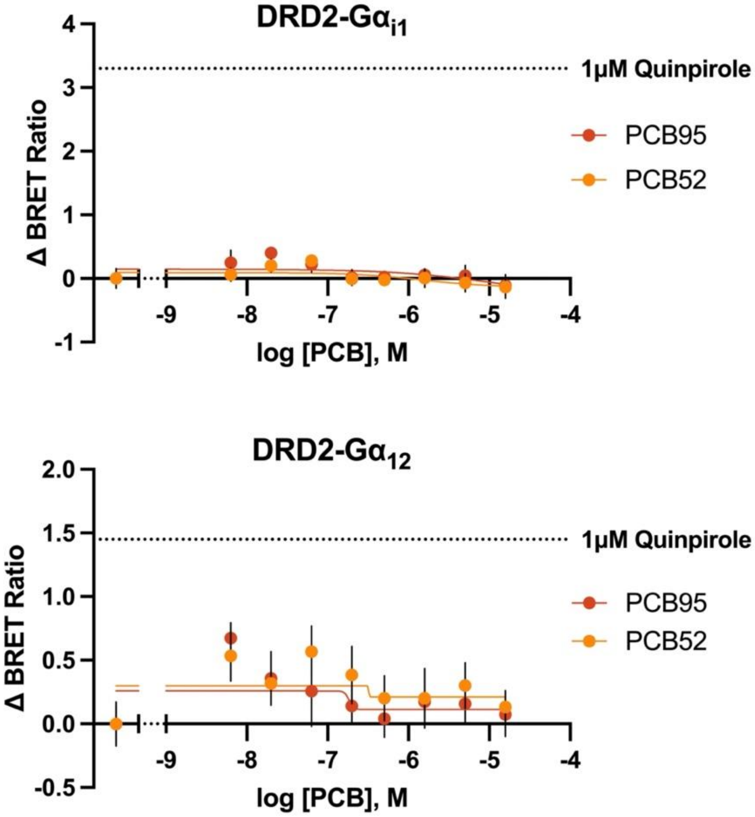

Figure S5 Concentration-response of PCB congeners against DRD2 in g protein association assays. Data points represent mean +/- SEM, in duplicate (n = 3).
